# Basal Cell Carcinoma of the Female Breast Masquerading as Invasive Primary Breast Carcinoma: An Uncommon Presentation Site

**DOI:** 10.1155/2018/5302185

**Published:** 2018-07-03

**Authors:** Mark B. Ulanja, Mohamed E. Taha, Arshad A. Al-Mashhadani, Marwah Muaad Al-Tekreeti, Christie Elliot, Santhosh Ambika

**Affiliations:** ^1^Department of Internal Medicine, University of Nevada Reno, School of Medicine, 1155 Mill Street, Reno, NV 89502, USA; ^2^American Public University System, 111 West Congress Street, Charles Town, WV 25414, USA

## Abstract

Skin cancer as a single entity is the most common malignancy in North America, accounting for half of all human cancers. It comprises two types: melanoma and nonmelanoma skin cancers. Of the nonmelanomas, basal cell carcinoma (BCC) constitutes about 80% of the cancers diagnosed every year. BCC usually occurs in sun-exposed areas such as the face and extremities. Occurrence in the nipple areolar complex is very rare. We present a case of a Caucasian woman who presented with what was initially thought to be invasive carcinoma of the breast involving the nipple areolar complex (NAC); however, the diagnosis was revealed to be a basal cell carcinoma after histopathological examination. The tumor was treated with modified radical mastectomy, with negative margins. The importance of this case lies in the rare site of presentation of basal cell carcinoma and the importance of early detection.

## 1. Introduction

Skin cancer as a single entity is the most common malignancy in North America [1]. They account for half of all human cancers. Generally, they could be divided into two types: melanoma and nonmelanoma skin cancers. The nonmelanoma type, which is the most common form, includes basal cell carcinoma and squamous cell carcinoma. Of the 3.5 million cases of nonmelanoma skin cancer (NMSC) diagnosed each year, 80% are basal cell carcinomas (BCCs), which makes BCCs the most common skin cancer [2]. It is most common among fair-skinned persons, with a lifetime risk of 33% to 39% in white men and 23% to 28% in white women in the United States [1].

The most important environmental risk factor is ultraviolet (UV) light exposure, hence BCC usually occurs in sun-exposed areas [3]. The occurrence of BCC in unexposed areas such as in the nipple areolar complex (NAC) is very rare [4, 5]. We present a case of breast cancer in a Caucasian woman, involving the nipple areolar complex (NAC) which was initially thought to be invasive carcinoma of the breast, but was subsequently diagnosed to be BCC.

## 2. Case Report

A 64-year-old Caucasian female presented to our emergency department (ED) with a two-day history of bleeding from her left breast. She has had a slowly enlarging growth on her left breast for the past two years, which initially started as a small papular lesion in the nipple areolar complex. Most recently, the mass became ulcerated with active serous discharge; however, due to the lack of health insurance, the patient did not seek any medical attention. For the past two days prior to presentation, she developed significant bleeding and oozing from the ulcerated mass, forcing her to report to the ED. There was associated localized breast pain, but no weight loss, fever, nausea, vomiting, abdominal pain, back pain, abdominal pain, shortness of breath, cough, blurry vision, nor headaches.

She had no prior personal or family history of skin and breast cancers. She had no history of excessive exposure to sunlight, radiation exposure, arsenic ingestion, or a history of immunosuppression.

**Figure 1:**
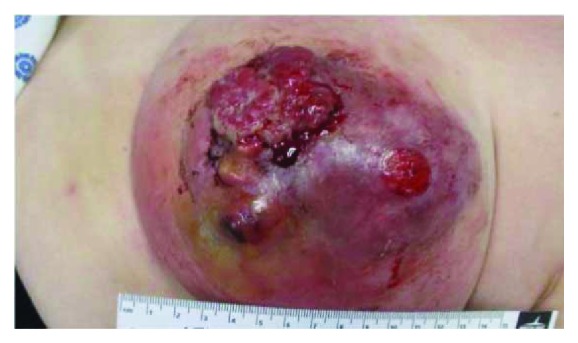
Left breast basal cell carcinoma showing ulcerations and bleeding.

Physical examination reveals an elderly female in no apparent distress. Vital signs were stable apart from an elevated blood pressure of 164/85 mmHg. Examination of the left breast revealed a large fungating mass of >10 cm in size, occupying most of the mid and outer breast with a distortion of the nipple areolar complex (Figure 1). There were several open wounds with active bleeding and a foul smell. The area of erythema was noted. There were palpable left axillary lymph nodes. The rest of the physical examination was unremarkable.

The provisional diagnosis was breast cancer with possible metastasis. Subsequently, the patient underwent workup to further characterize the mass and assess for metastasis. Computer tomography (CT) scan of the chest, abdomen, and pelvis was positive for a large, partially enhancing heterogeneous mass in the left breast and a calcified granuloma in the right lung field, in addition to mildly enlarged left axillary lymph nodes. No evidence of metastasis was identified in the abdomen and pelvis. Magnetic resonance imaging (MRI) of the brain with and without contrast was negative for brain lesions. There was no evidence of osseous metastatic disease as evident by the negative nuclear medicine bone scintigraphy.

**Figure 2:**
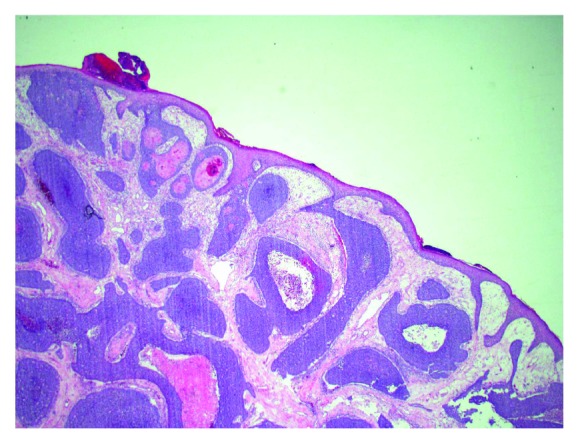
Histological findings on excisional biopsy H&E (hematoxylin and eosin stain) 2x, demonstrate nests of tumor cells arising from the surface epidermis.

**Figure 3:**
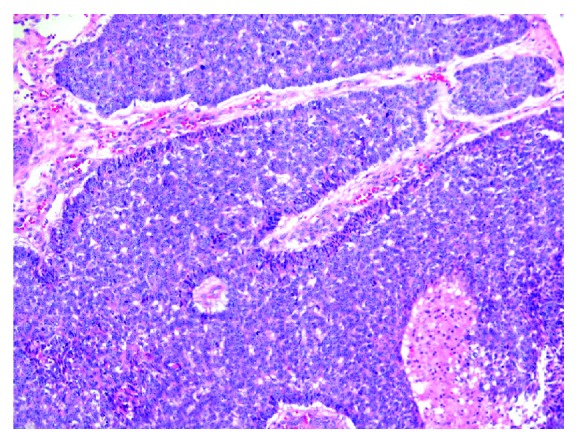
Histological findings on excisional biopsy H&E (hematoxylin and eosin stain) 10x show peripheral palisading of the tumor cells at the periphery of the nests.

**Figure 4:**
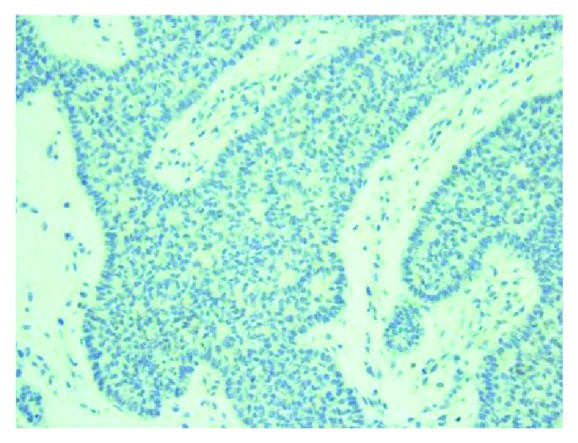
Immunohistochemical (IHC) stain 20x shows tumor cells to be negative for GATA3 (note: positive in breast primary).

**Figure 5:**
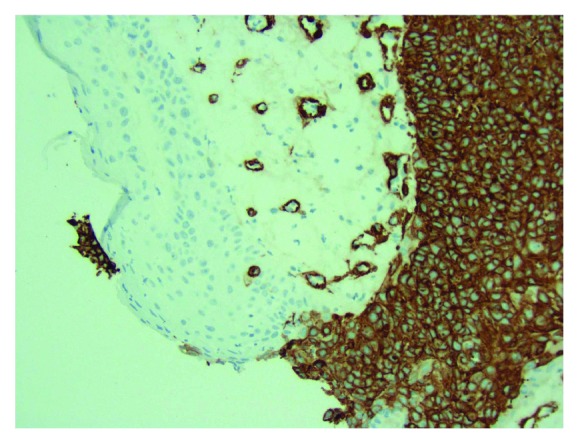
IHC stain (SMA—smooth muscle actin) 20x shows the normal epidermis to be negative (which is what is expected), but the tumor cells show strong cytoplasmic positivity. The circles that are also staining is smooth muscle in normal blood vessels (positive internal control).

Trucut excisional biopsy of the mass was performed. The initial histopathological exam was suggestive of an epidermal origin of the cancerous cells, raising the possibility of an adnexal primary such as basal cell carcinoma (Figures 2 and 3). Immunohistochemical (IHC) profile also favored a primary skin disorder over a breast primary (Figures 4 and 5). Utilizing NeoGenomics®, the cells were consistent with cutaneous basal cell carcinoma.

Post diagnosis, the patient underwent left modified radical mastectomy with axillary lymph node dissection. After histopathological exam for the dissected tissue and lymph nodes, a final diagnosis of invasive cutaneous basal cell carcinoma was made. The margins were tested negative for carcinoma. All the dissected 16 lymph nodes were negative for cancer. Subsequent treatment and oncological follow-up were scheduled with oncology.

## 3. Discussion

Basal cell carcinomas (BCCs) are nonmelanoma skin cancers arising from the basal layer of the epidermis and its appendages. They constitute eighty percent of skin cancers. They are slow-growing tumors and very rarely metastasize; however, if left untreated, they tend to grow and invade nearby tissues [6].

Geographically, there is profound variation in the incidence of BCCs due to the effect of ultraviolet light on its development. In the USA for instance, in 1990, the incidence of BCCs in the states which are in close proximity to the equator, like Hawaii, was twice as that of the Midwestern regions [7, 8].

The most important risk factor for BCCs is ultraviolet (UV) light exposure, particularly intermittent, intense UVB light exposure; hence, BCCs most commonly occur in sun-exposed areas [3]. Other risk factors include radiation therapy, chronic arsenic exposure, and long-term immunosuppression. In patients with early-onset or numerous BCCs, a syndromic manifestation of a genetic cause (e.g., basal cell nevus syndrome) should be considered [2].

The occurrence of BCC in the skin of the breast such as the nipple areolar complex (NAC) is very rare [4, 5]. It was first reported in 1893 [9] and as of September 2016, BCCs of the areolar and nipple have been described in 55 individuals of which 35 were males and 20 were females and the onset age ranged from 35 to 86 years [10, 11]. In a study by Betti et al., they found that 74% of the BCCs were located on the head and neck area, 26% were involved in the covered sites of the body, and only two cancers were involved in the nipple and areolar [10]. A histogenic relationship has been noted between pilosebaceous units and the development of BCC [12]. The NAC is deficient in pilosebaceous units and this may explain the paucity of BCCs in this area [13].

In regard to the etiology of BCC in the NAC, some studies have suggested that ultraviolet (UV) irradiation might be the main etiological factor. In one study, a history of extensive sun exposure was evident in three out of six cases with multiple BCC lesions in the NAC [13, 14]. Other potential risk factors include genetic predisposition, immunosuppression, ionizing radiation exposure, arsenic exposure, injuries such as burns or trauma, light-colored skin, previous BCCs at another site, and sunburns [15]. Similar to the majority of the cases of BCC, as well as in our case, no history of risk factors is identified.

Differential diagnosis of a BCC lesion in the NAC includes Paget's disease, eczema, adenoma of the nipple, papilloma of lactiferous ducts, syringomatous adenoma, invasive ductal carcinoma, and melanoma. Therefore, it is crucial to perform histopathological examination to establish the diagnosis [5]. In the NAC, BCC is considered to behave more aggressively than other anatomical sites, but other nonaggressive histological subtypes exist, and tumor recurrence is uncommon after the successful treatment of the primary cancer [15, 16]. In a previous study, a report of 3 out of 31 cases of BCC in the NAC have developed apparent axillary lymphadenopathy with histologically confirmed cases [15]. Takeno et al. found that axillary lymph node metastasis of basal cell carcinoma was about 11.5% in 26 patients [16], which was apparently high compared to the rate of 0.01–0.028% [17] noted by Elder et al. The likely explanation is that the subareolar plexus is rich in a network of lymphatic capillaries and this might provide high potential for metastasis of tumors in this area, hence this relative difference [16, 17].

Varying modalities of treatment are available for BCC in the NAC depending on the characteristics of the lesion. Options include medical treatment, photodynamic therapy, laser therapy, Mohs' microsurgery, and simple surgical excision with or without radiotherapy, as well as partial mastectomy with axillary dissection and surgical reconstruction of the breast [15, 16, 18]. This patient presented late because of the lack of health insurance, and oozing and bleeding were noted from the extensive ulcerated breast lesions. The initial histopathology report was not conclusive and had to be sent for further consultation. It was recommended that given the clinical impression and initial inconclusive histopathology report, mastectomy was most appropriate. It was conceivable that the patient will likely have persistently positive resection margins if reasonable attempts at excision and reexcision was made [19]. Also based on the relatively high incidence of maxillary lymph node metastasis [16], mastectomy was recommended as the best mode of treatment.

Our case presented with a 2-year history of a slowly growing papular lesion in the breast not associated with systemic symptoms except for local breast pain when it began to ulcerate, involving most part of the left breast. It is important to be aware that BCCs can become locally aggressive, without systemic symptoms. Simple mastectomy with left axillary lymph node dissection was performed. Lymph nodes were negative as well as the rest of the metastatic workup. Regular follow-up is very important to assess for recurrence or late manifestation that might develop from micro metastasis. Unfortunately, follow-up was difficult to establish due to health insurance constraints, but the patient was educated thoroughly regarding the examination of her skin and features of recurrence and advised regarding seeking medical help as early as possible.

## 4. Conclusion

For the past 125 years, only about 62 cases of BCC of the NAC have been reported, which highlights the rarity of this presentation. Clinicians should be aware of the occurrence of BCC in this unexposed region and should consider BCC as a differential diagnosis to other benign and malignant disorders affecting the NAC. Furthermore, given the rich lymphatic nature of the NAC, this cancer has the high potential for distant metastasis, and hence it is of great importance to be recognized early enough to institute appropriate treatment.
